# Microbial Community Structures and Methanogenic Functions in Wetland Peat Soils

**DOI:** 10.1264/jsme2.ME22004

**Published:** 2022-07-15

**Authors:** Wipoo Prasitwuttisak, Yuki Hoshiko, Toshinari Maeda, Akira Haraguchi, Katsunori Yanagawa

**Affiliations:** 1 Faculty of Environmental Engineering, The University of Kitakyushu, Kitakyushu 808–0135, Japan; 2 Department of Biological Functions Engineering, Graduate School of Life Sciences and Systems Engineering, Kyushu Institute of Technology, Kitakyushu, 808–0196, Japan

**Keywords:** wetland, methanogenesis, *mcrA*, *Candidatus* Bathyarchaeia

## Abstract

Methane metabolism in wetlands involves diverse groups of bacteria and archaea, which are responsible for the biological decomposition of organic matter under certain anoxic conditions. Recent advances in environmental omics revealed the phylogenetic diversity of novel microbial lineages, which have not been previously placed in the traditional tree of life. The present study aimed to verify the key players in methane production, either well-known archaeal members or recently identified lineages, in peat soils collected from wetland areas in Japan. Based on an ana­lysis of microbial communities using 16S rRNA gene sequencing and the mole­cular cloning of the functional gene, *mcrA*, a marker gene for methanogenesis, methanogenic archaea belonging to *Methanomicrobiales*, *Methanosarcinales*, *Methanobacteriales*, and *Methanomassiliicoccales* were detected in anoxic peat soils, suggesting the potential of CH_4_ production in this natural wetland. “*Candidatus* Bathyarchaeia”, archaea with vast metabolic capabilities that is widespread in anoxic environments, was abundant in subsurface peat soils (up to 96% of the archaeal community) based on microbial gene quantification by qPCR. These results emphasize the importance of discovering archaea members outside of traditional methanogenic lineages that may have significant functions in the wetland biogeochemical cycle.

Methane (CH_4_) is an important greenhouse gas due to its potent heat-trapping ability in the atmosphere. Although the quantity of CH_4_ in the atmosphere is lower than that of CO_2_, it may have a serious impact on the Earth’s climate. Increases in atmospheric methane occurred between 1982 and 2000 (Turner *et al.*, 2019). In 2007, the amount of atmospheric CH_4_ began to rapidly increase again after remaining stable for seven years (Schaefer *et al.*, 2016), which may be explained by a rise in emissions from natural wetlands and fossil fuel operations (Kirschke *et al.*, 2013). As a result, atmospheric CH_4_ has become a critical concern. In addition, it has garnered scientific interest and prompted the development of international treaty goals (Fletcher and Schaefer, 2019). The average global emission of CH_4_ is estimated to be 548 Tg year^–1^ (Kirschke *et al.*, 2013). Since approximately one-third of emissions is derived from natural wetlands, these environments are the largest natural source of the atmospheric CH_4_ budget (Dlugokencky *et al.*, 2011). CH_4_ is emitted from various types of anaerobic terrestrial and aquatic environments, such as rice paddies, freshwater sediments, organic waste deposits (landfills and sewage treatments), animal digestive tracts, and natural wetlands (Kirschke *et al.*, 2013). Natural wetlands are regarded as important terrestrial carbon reservoirs that may significantly respond to the global biogeochemical cycle and nutrient fluxes (Gorham, 1991).

Biogenic CH_4_ is generally derived from the anaerobic fermentation of organic matter and subsequent methanogenesis under thermodynamically favorable conditions for anaerobic bacteria and methane-producing archaea. Methanogenic archaea utilize a limited number of substrates to generate CH_4_ as the end product of anaerobic respiration (Liu and Whitman, 2008). To date, cultivated methanogenic archaea are considered to be restricted to only nine orders within three phyla (based on the GTDB taxonomic classification), in which there is at least one single pure culture representative (Lyu *et al.*, 2018; Rinke *et al.*, 2021). The five methanogenic orders in the phylum *Halobacteriota* are *Methanomicrobiales*, *Methanonatronarchaeales*, *Methanocellales*, *Methanotrichales*, and *Methanosarcinales*. *Methanobacteriales*, *Methanopyrales*, and *Methanococcales* belong to the phylum *Methanobacteriota*. The last order, *Methanomassiliicoccales*, is assigned to the phylum Thermoplasmatota. In addition, novel methanogenic lineages, including “*Candidatus* Methanofastidiosales”, “*Ca.* Methanomethylicia” (also known as Verstraetearchaeota), and “*Ca.* Bathyarchaeia” (previously known as MCG or the candidate phylum “*Bathyarchaeota*”) were recently revealed based on metagenomic information on environmental samples associated with CH_4_ metabolism (Evans *et al.*, 2015; Nobu *et al.*, 2016; Vanwonterghem *et al.*, 2016). Among these archaeal lineages, only five (*Methanobacteriales*, *Methanomicrobiales*, *Methanocellales*, *Methanosarcinales*, and *Methanomassiliicoccales*) are commonly found in peatlands (one of the wetland types) (Bräuer *et al.*, 2020).

In most studies, the production of CH_4_ through microbial methanogenesis has been examined by targeting functional genes corresponding to the presence of methanogenic communities. Cultivation-independent techniques, such as next-generation sequencing (16S rRNA gene-based) and phylogenetic construction, have been widely used to directly investigate microbial diversity and reveal distinct bacterial and archaeal branches with high efficiency in various environmental samples (Hillis, 1997; Karst *et al.*, 2018). Additionally, the functional *mcrA* gene, encoding the alpha subunit of methyl-coenzyme M reductase (MCR), is frequently used as a mole­cular marker to detect MCR-containing methanogenic archaea (Friedrich, 2005). The methanogenic community and physiological ecology have been extensively examined in the Northern peatlands, an important source of atmospheric methane and a large reservoir of terrestrial carbon (Basiliko *et al.*, 2003; Galand *et al.*, 2003; Yavitt *et al.*, 2012; Abdalla *et al.*, 2016). In the present study, we focused on the methanogenic community in the peat soils of Bogatsuru mire in Oita Prefecture (Kyushu, Japan). The accumulation of peat material in the wetlands induces a transition from a low to high moor, corresponding to the shift from nutrient-rich to nutrient-poor conditions. The intermediate moor harbors nutritionally diverse environments, which are expected to enable the formation of ecological niches for diverse microorganisms. Bogatsuru mire was listed as the largest intermediate moor in Japan when combined with Tadewara mire, and was designated as a Ramsar site in 2005. Nevertheless, the Bogatsuru microbial community has never been investigated. Therefore, based on 16S rRNA gene amplicon sequencing and functional gene targeting, we herein attempted to identify key methanogenic archaea in the acidic wetland, either well-established methanogens or novel lineages, in order to elucidate their phylogenetic distribution and metabolic functions.

## Materials and Methods

### Sampling site and sample collection

On 21 July 2020, peat soil samples were vertically collected from Bogatsuru mire (33°05′47.3″ N, 131°15′35.2″ E) using a Tomas-type peat sampler (Nose Tekkosho, Okayama, Japan) at three depths from the surface (10, 45, and 90‍ ‍cm) to cover aerobic and anaerobic representatives, designated as BO10, BO45, and BO90, respectively. The abundant species of vegetation at the sampling site were *Phragmites australis* (Cav.) Trin. ex Steud., *Moliniopsis japonica* (Hackel) Hayata, *Juncus effusus* L. var. *decipiens* Buchen, *Sphagnum fimbriatum* Wils., and *S. palustre* L. The atmospheric temperature at the time of sampling was 26°C. Water on the surface of the peatland was collected using a sterile syringe to perform on-site measurements of pH and the oxidation-reduction potential (ORP) with a portable meter (D-52, Horiba). In the soil CH_4_ gas ana­lysis, 2‍ ‍g of a soil sample was fixed on-site with mixed solutions containing 0.5‍ ‍mL of 10% benzalkonium chloride and 9.5‍ ‍mL of saturated sodium chloride solution in 20-‍mL vials sealed with headspace caps (Agilent). All samples were transferred to the laboratory on the same day. Samples for chemical and mole­cular biological ana­lyses were stored at 4 and –80°C, respectively, until further processing.

### Soil gas ana­lysis and water chemical composition

To quantify CH_4_ potentially produced from peat soils, 1‍ ‍mL of the headspace was analyzed using a gas chromatograph (Agilent) equipped with a MICROPACKED ST column (Shinwa Chemical Industries) and flame ionization detection (FID). The temperatures of the column, injector, and detector were 80, 100, and 300°C, respectively. The detection limit was 1 ppm. Regarding water geochemistry, water samples were filtered using a 0.2-μm single-use cellulose acetate membrane filter (Sartorius Stedim Biotech) prior to the quantification of ion concentrations. Cations (Ca^2+^, Fe^3+^, K^+^, Mg^2+^, and Na^+^) were analyzed using ICP-AES (ICPE-9800; Shimadzu), while anions (F^–^, Cl^–^, NO_3_^–^, SO_4_^2–^) were examined using an ion chromatograph (Dionex DX-120; Thermo Fisher Scientific). Acetate was analyzed using ion chromatography (Dionex ICS-2100; Thermo Fisher Scientific).

### Microbial community structure ana­lysis based on the 16S rRNA gene

The prokaryotic DNA of peat soil samples (BO10, B045, and BO90) was extracted using the DNeasy PowerSoil Kit (Qiagen), according to the manufacturer’s instructions. During extraction, microbial cells were mechanically disrupted using a μT-01 bead crusher (TAITEC). All extracted DNA samples were kept at –‍80°C for further ana­lyses. Purified genomic DNA was used to construct a PCR library of the hypervariable V3–V4 region of the 16S rRNA gene using the primers Bakt_341F and Bakt_805R, which were designed by Herlemann *et al.* (2011), and its taxonomic lineage was evaluated by Klindworth *et al.* (2013). Amplification was performed in a 30-μL reaction volume with initial denaturation at 98°C for 2‍ ‍min, 40 cycles of denaturation at 98°C for 30‍ ‍s, annealing at 55°C for 30‍ ‍s, elongation at 68°C for 30‍ ‍s, and final elongation at 68°C for 5‍ ‍min. PCR products were purified and processed as previously described (Yanagawa *et al.*, 2019). Sequencing was performed on the Illumina MiSeq platform. Microbiome ana­lyses, including quality filtering, sequence trimming, OTU clustering (97% cut-off), and taxonomic assigning, were processed using QIIME2 2018.2 (Bolyen *et al.*, 2019). The *Ca.* Bathyarchaeia sequences obtained from PCR amplicon sequencing were aligned with known representative sequences of *Ca.* Bathyarchaeia from previously reported genomic databases. A phylogenetic tree was constructed using the Maximum Likelihood method by RAxML 8.0 with the GTR GAMMA model in ARB software (Ludwig *et al.*, 2004). Bootstrap values were computed using 1,000 replicates. Raw sequence data were deposited in the Sequence Read Archive (SRA) under the accession number, DRA013094.

### Quantification of microbial 16S rRNA gene abundance

Prokaryotic 16S rRNA gene abundance was quantified using the Taqman probe-based qPCR method with the universal primer-probe set (Uni340F/Uni806R/Uni516F probe), the archaeal-specific primer-probe set (Arch349F/Arch806R/Arch516F probe), and the innuDry qPCR MasterMix Probe (Analytik Jena). Amplification was conducted in a 20-μL reaction volume with initial denaturation at 98°C for 2‍ ‍min, 50 cycles of denaturation at 98°C for 10‍ ‍s, annealing at 50°C (for the universal 16S rRNA gene) or 52°C (for the archaeal 16S rRNA gene) for 45‍ ‍s, and elongation at 72°C for 30 s. Targeted *mcrA* genes (marker genes for methanogenesis) were amplified using a specific primer set (ME3Mf/ME2′R) and MightyAmp for Real-Time PCR (TaKaRa Bio) under the following amplification conditions: initial denaturation at 94°C for 2‍ ‍min, 40 cycles of denaturation at 94°C for 40‍ ‍s, annealing at 52°C for 30‍ ‍s, and elongation at 68°C for 1‍ ‍min. *Ca.* Bathyarchaeia gene fragments were amplified using MightyAmp for Real-Time (TaKaRa Bio) and modified primers (MCG410F′/MCG528R′; Kubo *et al.*, 2012) under the following amplification conditions: initial denaturation at 95°C for 5‍ ‍min, 40 cycles of denaturation at 95°C for 10‍ ‍s, and combined annealing and elongation at 60°C for 45 s. Targeted gene abundance was quantified in triplicate using the real-time PCR system qTOWER^3^ G Touch (Analytik Jena). The non-specific amplification of targeted genes was confirmed by gel electrophoresis of the PCR product and a melting curve (for the *mcrA* gene). Details of the primers and probes used for qPCR are provided in Table S1.

### Molecular phylogeny of the *mcrA* gene

PCR amplification of the *mcrA* gene was conducted using a specific primer set, as previously described for qPCR with KOD FX Neo (TOYOBO). After gel extraction and purification, amplified PCR products were cloned, sequenced, and aligned, as previously described (Yanagawa *et al.*, 2019). Molecular phylogenetic trees of the *mcrA* gene were constructed using the neighbor-joining method in ARB software (Ludwig *et al.*, 2004). Bootstrap values were computed using 1,000 replicates. *mcrA* gene sequences were deposited in the DDBJ/EMBL/GenBank databases under the accession numbers, LC662834–LC662856.

## Results and Discussion

### Surface water chemistry

Water collected at the sampling site was used to assess the ORP, pH, and ion content. Based on the pH value, peat soil was mildly acidic (pH 6.45). Furthermore, according to data from samples collected during the rainy season (a period between June and July with a high average precipitation of 250–300‍ ‍mm, according to the Japan Meteorological Agency) and the value obtained from previous field measurements (pH 4.78) on 28 November 2019, soil pH may have been slightly higher than expected due to the dilution effect of rainfall. The surface water redox potential was 204 mV, suggesting oxidizing conditions at the soil surface. The ion composition ana­lysis revealed that sulfate (307‍ ‍μM), nitrate (200‍ ‍μM), and Ca^2+^ (238‍ ‍μM) were major ions found in surface water. Other minor cations and anions detected in surface water are listed in Table 1. Calcium and 3 other base cations (Na^+^, Mg^2+^, and K^+^) are important components that generally form the majority of cation groups found in peat surface water (Bourbonniere, 2009). The level of calcium ions in the present study was slightly higher than the maximum concentrations in peat bogs in Canada and northern USA (170‍ ‍μM), northern and central Europe (125‍ ‍μM), and in subtropical peatlands in central China (73‍ ‍μM). The concentrations of other base cations in our ana­lysis were in the range measured in surface water from northern hemisphere bogs (Bourbonniere, 2009). Variations in major cations may be dependent on mineral rock fragments and the ion exchange capacity of Sphagnum plants (Verry, 1975; Sjörs and Gunnarsson, 2002). The presence of nitrate in surface peat water may be attributed to microbial nitrification under an elevated surface water temperature and higher pH (Freeman *et al.*, 1993; Whitfield *et al.*, 2010). Nitrate concentrations in surface water from Bogatsuru were higher than those reported from northern and central Europe peat bogs (maximum concentration of 39‍ ‍μM) (Bourbonniere, 2009). However, the contribution of and variations in nitrate contents in the present study warrant further study to establish whether biologically relevant or anthropogenic disturbances occurred. It is important to note that this research has been interpreted only from the surface water chemistry profile (at a time point); therefore, an ana­lysis of the physicochemical characteristics of subsurface waters will provide insights into more environmental features than the present limited information.

### Microbial community in the surface layer (BO10)

**Fig. 1.**
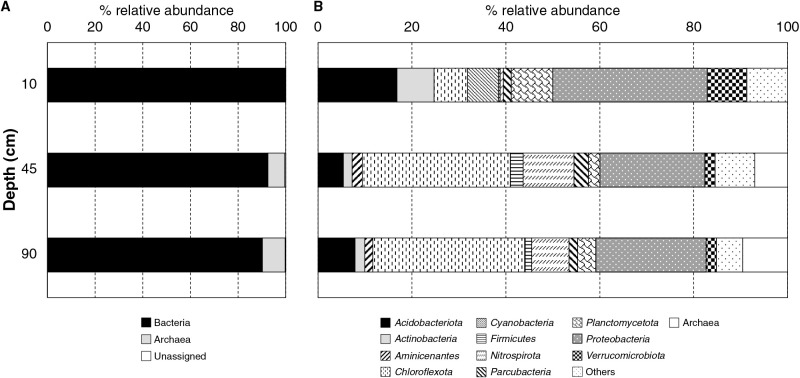
Microbial community compositions of peat soils based on a 16S rRNA gene amplicon ana­lysis using next-generation sequencing. (A) Domain level. (B) Bacterial diversity at the phylum level.

The microbial community composition of peat soils was analyzed using the 16S rRNA gene amplicon and next-generation sequencing platform. 16S rRNA gene sequencing lacks the representation of actual microbial abundance in samples due to the limitation of PCR amplification and sequencing (*e.g.*, the method of DNA extraction and purification, primer selection, and errors from sequencing technology) (Schloss *et al.*, 2011). In the present study, a total of 31,794 microbial sequences were obtained from peat soil samples (8,237 reads from BO10, 10,702 reads from BO45, and 12,855 reads from BO90) with an average length of 464 bp. The taxonomic classification and relative abundance of microorganisms are summarized in Fig. 1. At the domain level, bacterial sequences were dominant at all depths. Taxonomic classification at the phylum level revealed that members of the phyla, *Proteobacteria*, *Acidobacteriota*, *Planctomycetota*, and *Cyanobacteria*, were dominant in surface peat soil (Fig. 1B).

The water ana­lysis revealed that a higher nitrate content (200‍ ‍μM) was detected than the analytical range of surface peat waters (0.3–39‍ ‍μM) in northern peatlands (Bourbonniere, 2009), indicating the availability of nitrogen-transforming reactions in the Bogatsuru habitat. Microbial nitrogen-transformation pathways (*e.g.*, nitrogen fixation, nitrification, and denitrification) involve diverse groups of microorganisms. Based on the microbial community profile and taxonomic classification, several groups of bacteria associated with nitrogen fixation and transformation were identified in this study. According to taxonomic characterization at lower levels, the bacterial sequences of the orders *Rhizobiales* and *Planctomycetales* were detected at the highest proportion in surface peat soil (BO10). These bacterial groups have been reported to play a functional role in nitrogen cycling. *Rhizobiales* (*Bradyrhizobium* spp.) are nitrogen-fixing bacteria that generally live symbiotically with plant legumes (Kuypers *et al.*, 2018). Some members of *Planctomycetota* oxidize ammonium anaerobically using nitrite as an electron acceptor (Fuerst, 2005; Fuerst and Sagulenko, 2011). *Cyanobacteria*, which were only detected in BO10, have also been shown to assimilate nitrogen for growth through nitrogenase catalysis (Berman-Frank *et al.*, 2003; Bothe *et al.*, 2010).

### Microbial community in middle and deep layers (BO45 and BO90)

Based on the results of the soil gas ana­lysis, CH_4_ was only detected in BO45 (0.27±0.14‍ ‍mM). By referring to 16S rRNA gene amplicon sequencing, archaeal sequences were detected in BO45 and BO90 (Fig. 1A). The community compositions of peat soil at depths of 45 and 90‍ ‍cm were slightly different from those of surface samples, mainly *Anaerolineales*, *Nitrospirales*, *Syntrophobacterales*, and the candidate order GIF9, which were detected at a higher proportion (>500 sequence read counts) than in surface soil. Bacteria from the genus *Nitrospira* (belonging to the phylum *Nitrospirota*) were recently discovered to undergo complete ammonia oxidation (comammox) (Daims *et al.*, 2015; van Kessel *et al.*, 2015). Furthermore, diverse clades of comammox *Nitrospira* have been detected in the sediment along estuarine tidal flat wetlands (Sun *et al.*, 2020). *Nitrospira* members were dominant in the low dissolved oxygen reactor of a wastewater treatment system (Roots *et al.*, 2019) and were proven to oxidize formate using nitrate as an electron acceptor under anoxic conditions (Koch *et al.*, 2015).

Other major bacterial sequences detected in subsurface peats were affiliated to the phylum *Chloroflexota* (Fig. 1B). Members of *Chloroflexota* have been identified in sediments and are suggested to be involved in the subsurface carbon cycle (Blazejak and Schippers, 2010; Kadnikov *et al.*, 2012). The metabolic lifestyles of *Chloroflexota* in sediments retrieved from genomic ana­lyses include sugar and amino acid degradation, acetate utilization, and nitrate respiration and nitrification (Hug *et al.*, 2013).

The sequences of well-known methanogenic archaea in the orders *Methanomicrobiales* and *Methanosarcinales* were detected at a depth of 45‍ ‍cm. *Thermoplasmatales* were also identified in subsurface peats (BO45 and BO90). In addition, *Ca.* Bathyarchaeia sequences were more abundant in BO45 and BO90 than other archaeal sequences. Other archaeal groups, including *Iainarchaeota*, *Hadarchaeia*, *Nitrososphaeria*, and *Nanoarchaeota*, were also detected in subsurface peat soils.

### Microbial gene abundance

The distribution of microbial gene numbers along the vertical soil depth (Table 2) was quantified by qPCR using specific primer sets. Prokaryotic 16S rRNA gene numbers ranged between 2.56×10^8^ and 8.73×10^8^ genes g^–1^ peat. The archaeal 16S rRNA gene number was lower than those of the prokaryotes at all soil depths, ranging between 2.81×10^6^ and 2.58×10^7^ genes g^–1^ peat. The abundance of archaeal genes was the highest in the middle depth layer. Furthermore, the ratio of archaeal 16S rRNA genes to prokaryotic 16S rRNA genes ranged between 0.4% and 2.9%, suggesting the low abundance of archaea at all depths.

The abundance of the *mcrA* gene was interpreted based on a qPCR data ana­lysis and gel-electrophoresis confirmation (Fig. S2), and *mcrA* genes were only detected in subsurface soils (45 and 90‍ ‍cm) with the highest copy number of 3.91×10^6^ genes g^–1^ peat at a depth of 45‍ ‍cm. *mcrA* gene numbers were higher than those previously observed at subsurface peats (ranging from 10^4^–10^5^ genes g^–1^ peat) in Japanese wetlands (Akiyama *et al.*, 2011). If we assume that archaea and methanogens carry one copy of the 16S rRNA and *mcrA* genes, respectively (Kembel *et al.*, 2012; Louca *et al.*, 2018), MCR-containing archaea in the present study may have accounted for approximately 15% of the archaeal sequences at a depth of 45‍ ‍cm. Furthermore, the high copy number of the *mcrA* gene corresponded with the detection of CH_4_ at a depth of 45‍ ‍cm from the soil gas ana­lysis, suggesting the production potential of CH_4_ from methanogenic archaea.

*Ca.* Bathyarchaeia 16S rRNA genes were detected at depths of 45 and 90‍ ‍cm using the modified primers, with copy numbers of 4.45×10^6^ and 4.59×10^6^ genes g^–1^ peat, respectively. If we assume that the copy number of the 16S rRNA gene of *Ca.* Bathyarchaeia is equal to 1, the ratios of *Ca.* Bathyarchaeia to archaea in BO45 and BO90 were 18 and 97%, respectively, indicating the distribution of *Ca.* Bathyarchaeia in the archaeal community in the Bogatsuru wetland. Microbial gene abundance is summarized in Table 2.

### Phylogenetic composition of the *mcrA* gene

**Fig. 2.**
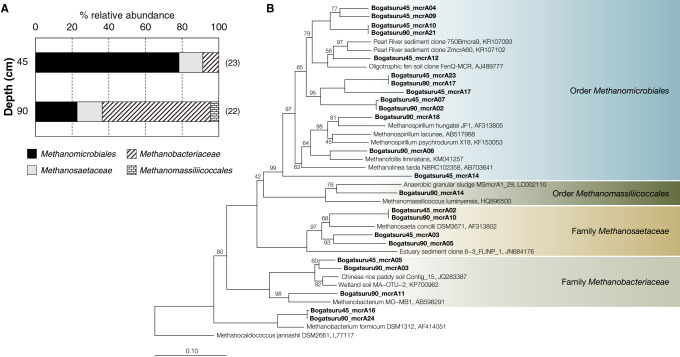
(A) Relative abundance of *mcrA* phylotypes. The number of *mcrA* clones is indicated in the brackets. (B) Molecular phylogenetic tree of *mcrA* gene sequences detected in peat soils constructed by the neighbor-joining method. Bootstrap values were computed with 1,000 replicates. The sequences obtained in the present study are indicated in bold characters covering 2 orders and 2 families of MCR-containing methanogenic archaea. The scale bar indicates the number of substitutions per site.

The phylogenetic diversity of *mcrA* was assessed using mole­cular cloning. The taxonomic classification of *mcrA* sequences is shown in Fig. 2A, while the phylogenetic tree is shown in Fig. 2B. A total of 23 and 22 clones were obtained in the *mcrA* clone library of BO45 and BO90 samples, respectively. Based on the results obtained, most of the *mcrA* nucleotide sequences in the BO45 library were phylogenetically classified into *Methanomicrobiales*, which accounted for approximately 78% of all *mcrA* clone sequences. *Methanobateriaceae* *mcrA* was dominant in BO90, comprising approximately 59% of all sequences. The methanogenic lineage in *Methanosaetaceae* was detected as a minority group at depths of 45 and 90‍ ‍cm. This result corresponded with previous finding showing the dominance of *Methanomicrobiales* in wetlands in Hokkaido, followed by a small proportion of *Methanosaetaceae* (Narihiro *et al.*, 2011). *Methanomassiliicoccales* accounted for 9% of all *mcrA* clones in the deepest peat soil (BO90).

### Phylogenetic composition and metabolic potential of *Ca.* Bathyarchaeia

**Fig. 3.**
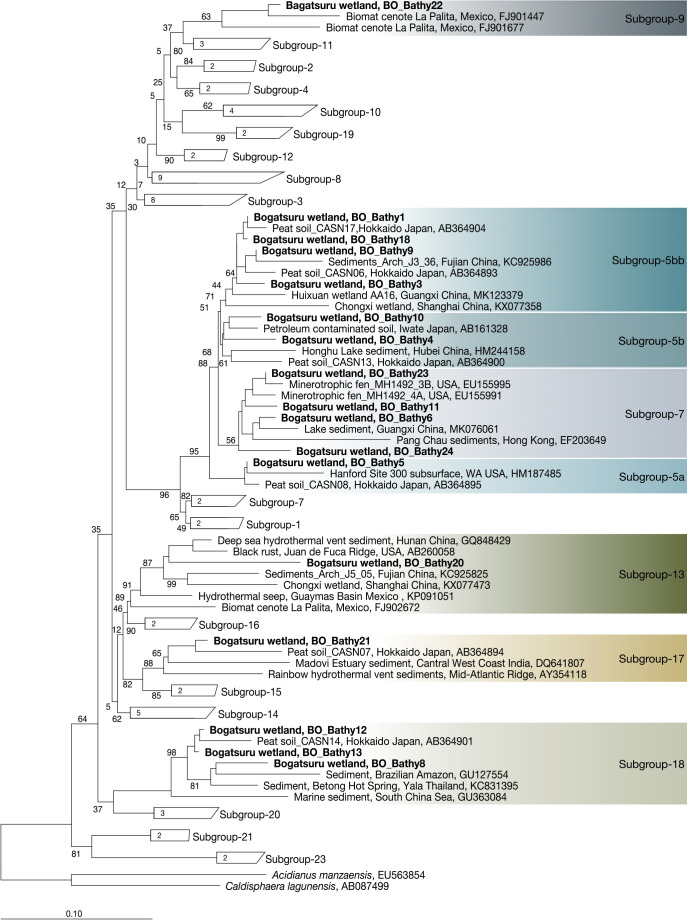
Molecular phylogenetic tree of *Candidatus* Bathyarchaeia 16S rRNA gene sequences detected in peat soils constructed by the Maximum Likelihood method. Bootstrap values were computed with 1,000 replicates. The sequences obtained in the present study are indicated in bold characters spanning the *Ca.* Bathyarchaeia subgroups. The names of *Ca.* Bathyarchaeia subgroups are based on Zhou *et al.* (2018). The scale bar indicates the number of substitutions per site.

*Ca.* Bathyarchaeia sequences obtained from the 16S rRNA gene amplicon ana­lysis were aligned and affiliated with the phylogenetic tree of archaea (Fig. 3). Based on the phylogenetic ana­lysis, *Ca.* Bathyarchaeia detected in the present study belonged to various subgroups (Subgroup-5a, 5b, 5bb, 7, 9, 13, 17, and 18) (Zhou *et al.*, 2018), indicating the diversity of this archaeal lineage in the terrestrial wetland ecosystem. *Ca.* Bathyarchaeia sequences have previously been detected in more than half of the archaeal populations in various peatlands (Rooney-Varga *et al.*, 2007; Hawkins *et al.*, 2014; Xiang *et al.*, 2017). Nevertheless, their ecological functions in peatland ecosystems have yet to be confirmed. Based on physiological and genomic characterizations, members of *Ca.* Bathyarchaeia possess diverse trophic and metabolic properties, including methanogenesis, and have been reported to utilize proteins, aromatic compounds, plant-derived carbohydrates, and lignin (Lloyd *et al.*, 2013; Meng *et al.*, 2014; Lazar *et al.*, 2016; Yu *et al.*, 2018). However, the *Ca.* Bathyarchaeia sequences retrieved herein deviated from the recognized methane-metabolizing groups BA1 (subgroup-3) and BA2 (subgroup-8), which have been proposed to encode methyl coenzyme M reductase (Evans *et al.*, 2015). Notably, qPCR quantification showed that the proportion of *Ca.* Bathyarchaeia was high in archaea, which positively encourages the need for further studies. Future research that focuses on the characterization of metabolic capability, particularly the confirmation of MCR-containing *Ca.* Bathyarchaeia, is warranted.

### Methanogenic potential and biogeochemical interaction in wetland soils

The *mcrA* gene phylotype revealed that members of *Methanomicrobiales*, which are well-recognized hydrogenotrophic methanogens that generally reduce CO_2_ to methane with H_2_ and/or formate as the electron donor, were mainly detected in the present study. They have been found in diverse anaerobic natural habitats, such as freshwater and marine sediments, rice paddies, animal digestive tracts, and wetlands (Jabłoński *et al.*, 2015). Known *Methanomicrobiales* representatives that have been successfully isolated from peat include *Methanosphaerula palustris* E1-9c (Cadillo-Quiroz *et al.*, 2008; Cadillo-Quiroz *et al.*, 2009) and *Methanoregula boonei* 6A8 (Bräuer *et al.*, 2006). The minor groups of methanogenic archaea present in peat soils were *Methanobacteriaceae* and *Methanoseataceae*. *Methanobacteriaceae* also perform CO_2_ reduction coupled with H_2_ oxidation for methanogenesis. However, *Methanoseataceae* are considered to be acetate utilizers, with acetate cleaved to form methane and carbon dioxide as terminal products through acetoclastic methanogenesis. Culture representatives of *Methanobacteriales* and *Methanosarcinales* are *Methanobacterium paludism* SWAN1 (Cadillo-Quiroz *et al.*, 2014) and *Methanothrix thermoacetophila* PT (Kamagata *et al.*, 1992), respectively. The order *Methanomassiliicoccales*, which was detected in the deepest soil in the present study, is an obligate methylotroph that produces methane from the reduction of methanol, methyl sulfide, and methylated amines (Paul *et al.*, 2012; Lang *et al.*, 2015). The representative of this order was initially isolated from human feces and called *Methanomassiliicoccus luminyensis* B10 (Dridi *et al.*, 2012). *Thermoplasmatales*, which are members of the class *Thermoplasmata*, were also detected based on 16S rRNA gene amplicon sequencing.

The detection of methanogenic archaea and a functional gene for methanogenesis in the present study suggested the potential of methane production using peat soils, either via hydrogenotrophic or acetoclastic methanogenesis. In contrast, net methane emission from marine sediments and terrestrial environments may be neutralized by anaerobic methanotrophic archaea (ANME) via the anaerobic oxidation of methane prior to its escape into the atmosphere (Knittel and Boetius, 2009). Based on 16S rRNA gene reads, no sequences of ANME were detected in the present study. Another group of microbes may also utilize methane aerobically, namely, aerobic methanotrophic bacteria (Dedysh and Knief, 2018). These organisms use methane monooxygenase to convert methane to methanol. Sequences of aerobic methanotrophic bacteria of the phylum *Verrucomicrobiota*, *Methylacidiphilum*, were detected at subsurface peats (BO45 and BO90). Genomic ana­lyses have shown that representative strains of *Methylacidiphilum* possess monooxygenase, similar to methanotrophs in the phylum *Proteobacteria*, which demonstrates a capability for aerobic methane oxidation (Dunfield *et al.*, 2007; Op den Camp *et al.*, 2009).

In anaerobic environments, methanogenic archaea compete with sulfate-reducing bacteria for available common substrates (Muyzer and Stams, 2008). Therefore, the presence of sulfate in such an environment is a key factor in trophic competition. In the present study, sulfate was detected based on the geochemistry of surface water (Table 1). The results of 16S rRNA gene amplicon sequencing revealed that the sulfate-reducing bacteria, *Desulfobacca*, affiliated with *Desulfobacterota*, were dominant in anoxic subsurface peats (BO45 and BO90), as depicted by the constitution of a relative high ratio of *Desulfobacterota* sequences in Fig. S1. *Desulfobacca* accounted for approximately 46 and 49% of all *Desulfobacterota* sequence reads in BO45 and BO90, respectively. Bacterial isolates belonging to the genus *Desulfobacca* have been isolated from granular sludge (Göker *et al.*, 2011). Furthermore, a physiological ana­lysis revealed that they utilized acetate as the sole carbon source and sulfate as an electron acceptor. Competition between sulfate reducers and acetoclastic methanogens for acetate utilization may occur in Bogatsuru wetlands because *Desulfobacca* and *Methanoseataceae* were detected in the present study.

Combined environmental omics approaches have been extensively proven as an advantageous strategy for identifying unknown microbial diversity, mainly in the context of examining key players in biogeochemical processes. Since methanogenic archaea in peatlands are difficult to culture due to, for example, their specific optimal growth requirements, potential syntrophic bacterial partners, environmental conditions, and generation time (Wolfe, 2011; Khelaifia *et al.*, 2013; Narihiro and Kamagata, 2013; Carson *et al.*, 2019), the recovery of genomic data may reveal taxonomic profiles and imply their functional properties. Further studies need to focus on the cultivation and isolation of uncultured methanogens and microbial syntrophs, which will contribute to our understanding of and reveal important information on microbial physiology and their functions that may provide feedback regarding the global methane and carbon cycle.

## Conclusions

In the present study, cultivation-independent mole­cular ana­lyses based on the 16S rRNA gene amplicon and functional *mcrA* gene were used to evaluate key microbial groups and their potential activities in metabolic methane production. Members of well-known methanogenic archaea were detected, which corresponded with the detection of the *mcrA* gene in anoxic subsurface peats. Members of *Ca.* Bathyarchaeia, the uncultivated archaea that are considered to play a role in biogeochemical cycles, were abundant in the present study. The results obtained prompt the further development of culturing innovations (culture-based experiments) and complete genomic characterization, which will be useful for providing comprehensive metabolic insights into *Ca.* Bathyarchaeia.

## Citation

Prasitwuttisak, W., Hoshiko, Y., Maeda, T., Haraguchi, A., and Yanagawa, K. (2022) Microbial Community Structures and Methanogenic Functions in Wetland Peat Soils. *Microbes Environ ***37**: ME22004.

https://doi.org/10.1264/jsme2.ME22004

## Supplementary Material

Supplementary Material

## Figures and Tables

**Table 1.** Water chemistry characteristics.

ORP (mV)	Acetate (μM)	K^+^ (μM)	Na^+^ (μM)	Ca^2+^ (μM)	Mg^2+^ (μM)	Fe^3+^ (μM)	F^–^ (μM)	Cl^–^ (μM)	NO_3_^–^ (μM)	SO_4_^2–^ (μM)
204	5.4	17	137	238	31	3	4	59	200	307

**Table 2.** Abundance of 16S rRNA and *mcrA* genes in peat soils assessed by qPCR.

Sample ID	Prokaryotic 16S rRNA (genes g^–1^ peat)	Archaeal 16S rRNA (genes g^–1^ peat)	*mcrA* (genes g^–1^ peat)	*Candidatus* Bathyarchaeia 16S rRNA (genes g^–1^ peat)
BO10	7.21±1.62×10^8^	2.81±1.92×10^6^	Not detected	Not detected
BO45	8.73±6.07×10^8^	2.58±0.22×10^7^	3.91±1.82×10^6^	4.59±1.07×10^6^
BO90	2.56±3.10×10^8^	4.64±1.11×10^6^	6.36±2.10×10^5^	4.45±0.99×10^6^

Data are shown as means±standard deviation.
