# Supplementary material for: Microbial Community Structures and Methanogenic Functions in Wetland Peat Soils
Source: Microbes Environ. 2022 Jul 15;37(3):ME22004. doi: 10.1264/jsme2.ME22004 (PMC9530717; doi:10.1264/jsme2.ME22004)
Supplement: Supplementary file 1 — Supplementary Material [file 37_22004_s1.pdf]

Table S1. List of primers and probes used for PCR and qPCR assays.

Target gene	Assay	Primer/Probe	Sequence (5'-3')	Reference
Prokaryotic 16S rRNA gene	qPCR	Uni340F<br>Uni806R<br>Uni516F (Taqman probe)	CCTACGGGRBGCASCAG<br>GGACTACNNGGGTATCTAAT<br>TGYCAGCMGCCGCGGTAAHACVNRS	Takai and Horikoshi 2000
Archaeal 16S rRNA gene	qPCR	Arch349F<br>Arch806R<br>Arch516F (Taqman probe)	GYGCASCAGKCGMGAAW<br>GGACTACVSGGGTATCTAAT<br>TGYCAGCCGCCGCGGTAAHACCVGC	Takai and Horikoshi 2000
Prokaryotic 16S rRNA gene	NGS	Bakt_341F<br>Bakt_805R	CCTACGGGNGGCWGCAG<br>GACTACHVGGGTATCTAATCC	Herlemann *et al*., 2010
mcrA gene	qPCR/Cloning	ME3MF<br>ME2'R	ATGTCNGGTGGHGTMGGSTTYAC<br>TCATBGCRTAGTTDGGRTAGT	Nunoura *et al*., 2008
Bathyarchaeia 16S rRNA gene	qPCR	MCG410F'<br>MCG528R'	WCCGCTGAGGDYGGCTTTT<br>CTCRGRGRGCTGGTATTACCG	Modified from Kubo *et al*. 2012

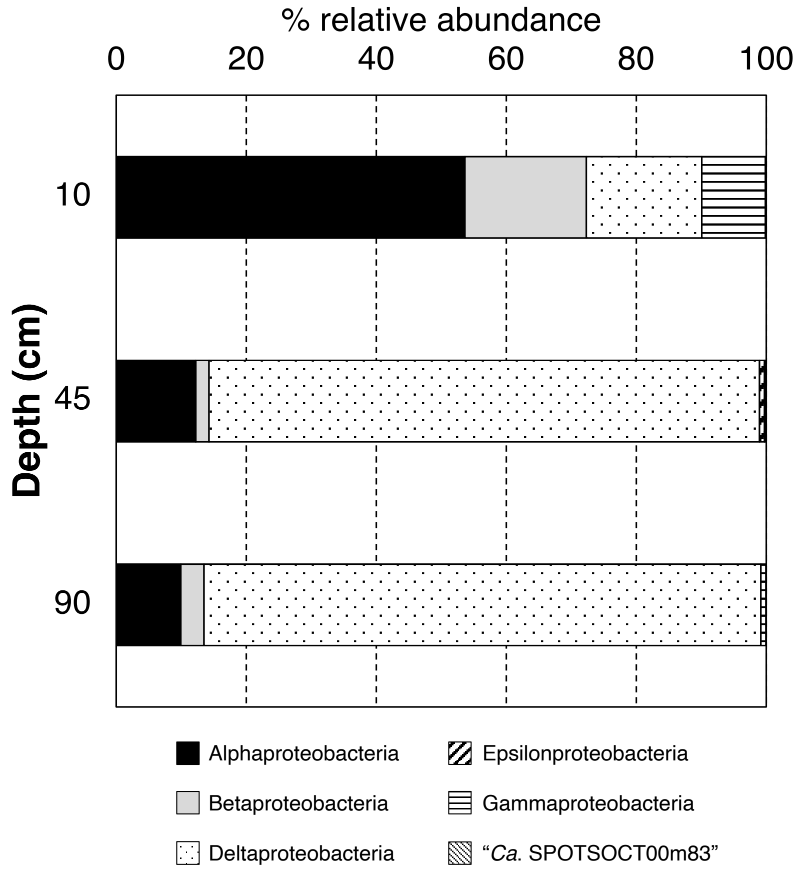

Fig. S1. Relative abundance ratio of the class-level groups of Proteobacteria in the peat soils.

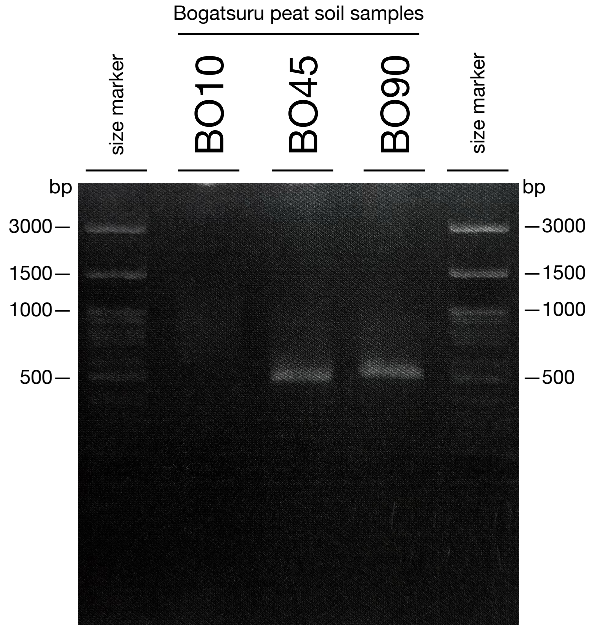

Fig. S2. Result of *mcrA* gene amplification from Bogatsuru peat soil samples for qPCR analysis. A molecular marker (GeneDirex, USA) is indicated at both sides of the sample lanes. The gel was made using 2% TAE agarose. PCR products (~ 500 bp) were loaded with Novel Juice (GeneDirex, USA) staining.

References

Herlemann, D.P.R., Labrenz, M., Jürgens, K., Bertilsson, S., Waniek, J.J., and Andersson, A.F. (2011) Transitions in bacterial communities along the 2000 km salinity gradient of the Baltic Sea. *ISME J* **5**: 1571–1579.

Kubo, K., Lloyd, K.G., Biddle, J.F., Amann, R., Teske, A., and Knittel, K. (2012) Archaea of the Miscellaneous Crenarchaeotal Group are abundant, diverse and widespread in marine sediments. *ISME J* **6**: 1949–1965.

Nunoura, T., Oida, H., Miyazaki, J., Miyashita, A., Imachi, H., and Takai, K. (2008) Quantification of mcrA by fluorescent PCR in methanogenic and methanotrophic microbial communities. *FEMS Microbiol Ecol* **64**: 240-247.

Takai, K., and Horikoshi, K. (2000) Rapid detection and quantification of members of the archaeal community by quantitative PCR using fluorogenic probes. *Appl Environ Microbiol* **66**: 5066-5072.
